# Regenerative Inflammation in IBD: How Type I Interferons and TNF Cross-Talk Converts Epithelial Repair into Therapeutic Response

**DOI:** 10.3390/cells15131144

**Published:** 2026-06-24

**Authors:** Emelia A. M. Hinton, Roslyn A. Kemp, Michael Schultz, Safina Gadeock

**Affiliations:** 1 Department of Microbiology and Immunology, Division of Health Sciences, University of Otago, Dunedin P.O. Box 56, New Zealand; hinem543@student.otago.ac.nz (E.A.M.H.); roslyn.kemp@otago.ac.nz (R.A.K.); 2 Department of Gastroenterology, Health New Zealand Southern, Dunedin 9054, New Zealand; michael.schultz@otago.ac.nz

**Keywords:** inflammatory bowel disease, TNF, type I interferons, epithelial regeneration, therapeutic resistance, epithelial memory, organoids, regenerative inflammation

## Abstract

**Highlights:**

**What are the main findings?**

- TNF is associated with both immune activation and epithelial regenerative responses and has been shown in experimental systems to influence progenitor cell expansion, lineage plasticity, and chemokine signaling in the intestinal epithelium.
- Chronic TNF exposure, in combination with type I interferon (IFN-I) signaling, may contribute to the persistence of epithelial regenerative programs and is associated with a sustained state of “regenerative inflammation” in preclinical and translational studies

**What are the implications of the main findings?**

- TNF-IFN-I associated epithelial reprogramming may contribute to sustained intestinal inflammation and could represent a mechanism supporting disease persistence beyond classical immune pathways.
- This form of epithelial imprinting may provide a potential mechanistic framework for anti-TNF non-response and highlights epithelial–immune crosstalk as a candidate area for therapeutic targeting in IBD, although clinical validation remains limited.

**Abstract:**

Inflammatory bowel diseases (IBD) are increasingly recognized as disorders in which epithelial dysfunction and maladaptive regeneration may be as important as immune dysregulation. Tumor necrosis factor (TNF), a key mediator of intestinal inflammation and a therapeutic target, plays a dual role in both immune activation and epithelial repair by regulating progenitor cell expansion, lineage plasticity, and chemokine signaling in the intestinal epithelium. During acute injury, TNF-associated responses are generally considered adaptive, supporting crypt repair, barrier restitution, and secretory remodeling pathways. However, in chronic disease, persistent TNF exposure, potentially reinforced by type I interferons (IFN-I), may contribute to the persistence of epithelial regenerative pathways. IFN-I signaling has been suggested in experimental and translational studies to reinforce chemokine networks and transcriptional imprinting. We propose that this potentially converts physiological repair into a sustained state of what we have termed “regenerative inflammation,” in which epithelial-derived signals may perpetuate immune recruitment and tissue remodeling. Such TNF-IFN-imprinted epithelial states may contribute to sustained pathology in a subset of patients and could be associated with reduced responsiveness to anti-TNF therapy, although direct causal evidence in human disease remains limited. By integrating mechanistic, organoid-based, and clinical observational evidence, we propose that chronic TNF–IFN crosstalk may contribute to a self-sustaining regenerative inflammatory circuit, providing a conceptual framework for disease persistence in IBD and highlighting potential opportunities to target epithelial-immune interactions.

## 1. Introduction

Inflammatory bowel diseases (IBD), including Crohn’s disease (CD) and ulcerative colitis, involve chronic intestinal inflammation, impaired mucosal repair and barrier function [1]. Although anti-TNF therapies such as infliximab and adalimumab, have significantly improved disease management, therapy fails for approximately 30–40% of patients, and responsiveness is often lost over time [2]. This therapeutic gap has prompted a re-evaluation of disease mechanisms beyond classical immune dysregulation, with increasing attention on the intestinal epithelium as a critical determinant of disease outcome [3]. The epithelium functions not only as a physical barrier but also as an active regulator of immune responses, integrating cytokine signals and coordinating tissue repair [3].

The intestinal epithelium maintains a remarkable capacity for regeneration, driven by a dynamic interplay between stem cells, niche cells, and the surrounding microenvironment [4]. This regenerative capacity is essential for effective epithelial repair following injury and restoration of barrier integrity. Tissue-resident stem cells at the crypt base respond to epithelial injury by initiating proliferation and differentiation pathways, while the niche secretome molecules, including Wingless related integration site (Wnt), R-spondin, and Notch ligands, coordinate spatial and temporal aspects of repair [4]. Regeneration is not solely a stem cell-intrinsic process; rather, it is comprehensively shaped by the surrounding mesenchymal, immune, and microbial cues in the microenvironment [5], which collectively determine whether repair proceeds toward homeostasis or becomes maladaptive. Signals such as inflammatory cytokines, microbial metabolites, and extracellular matrix components dynamically modulate epithelial fate decisions [5]. In physiological contexts, this plasticity allows the intestine to recover from transient insults [6]. However, in chronic inflammatory settings such as IBD, persistent immune cell activation disrupts the regenerative niche [4,6].

While TNF blockade remains central to IBD therapy, a significant subset of patients exhibits refractory disease, with anti-TNF non-response frequently associated with amplified IFN-I activity [7,8,9]. Together, TNF and IFN-I are increasingly implicated in driving a state of “regenerative inflammation,” in which tissue repair programs become reprogrammed to sustain inflammatory signaling rather than restore epithelial homeostasis [10,11,12,13,14]. In this context, elevated epithelial IFN-I signaling has been linked to persistent stress responses and impaired repair capacity [15,16,17], supporting a model in which TNF-IFN-I crosstalk within the epithelium and its niche shapes stem cell behavior and disrupts coordinated regeneration. This basis is being refined through emerging data that maps how dysregulated repair processes evolve into chronic inflammation, highlighting alterations in cytokine crosstalk, epithelial state plasticity, and stromal support [18,19]. Advances in single-cell transcriptomics and tissue-targeted in vivo and in vitro models have revealed fundamental alterations in epithelial states in IBD, including the emergence of regenerative, progenitor-like and inflammatory cell populations that persist during chronic disease [20,21,22,23,24,25]. These findings suggest that epithelial dysfunction is not merely a consequence of inflammation but a driver of disease chronicity. The central question remains: why do regenerative pathways fail to resolve in IBD? We suggest that sustained TNF-IFN-I signaling locks intestinal epithelial cells in a continuously activated state, shifting repair away from homeostatic mechanisms toward maladaptive pathways that reinforce chronic inflammation, defining regenerative inflammation. Understanding these mechanisms will be critical for identifying patients at risk of persistent disease and for developing therapies that restore mucosal homeostasis.

## 2. Epithelial Regeneration in Homeostasis and Inflammation

The intestinal epithelium undergoes rapid cellular turnover driven by Lgr5^+^ stem cells located at the crypt base [5,6]. Under homeostatic conditions, these stem cells give rise to transit amplifying (TA) progenitor cells, which proliferate rapidly and subsequently differentiate into absorptive enterocytes and secretory lineages, including goblet, Paneth (small intestine), M cells, tuft cells and enteroendocrine cells. TA cells form a key amplification zone linking rapidly dividing stem cells to a committed differentiated progeny, enabling high epithelial turnover while preserving stem cell integrity [5,6]. Importantly, the TA cell compartment not only functions as a proliferative intermediate in homeostasis but also represents a key site where features of regenerative inflammation emerge, contributing also to the cellular pathways that support the formation of wound-associated epithelium (WAE) during injury-driven repair [5,6,9].

Upon injury, the epithelium activates highly plastic regenerative pathways that drive the formation of WAE, a transient repair state that emerges at sites of epithelial disruption to restore barrier integrity [6,12]. WAE is characterized by a flattened, migratory, de-differentiated epithelial phenotype enriched for repair and stress response genes, including Keratin 6 (KRT6), Keratin 17 (KRT17), Clusterin (CLU), Stem cell antigen 1 (SCA1), Tumor associated calcium signal transducer 2 or Trophoblast cell surface antigen 2 (TACSTD2 or TROP2), and Annexin A1 (ANXA1), alongside chemokines such as chemokine (C-X-C motif) ligand 1 (CXCL1), 10 (CXCL10) and 11 (CXCL11) [12,19,26]. These features reflect a transient epithelial program that prioritizes migration, survival, and barrier reconstitution over absorptive or secretory function. Studies have demonstrated that this state is coordinated by transcriptional regulatory pathways such as Yes-associated protein/Transcriptional coactivator with PDZ-binding motif (YAP/TAZ), and inflammatory inputs including TNF and interleukin (IL)6 family cytokines, while emerging evidence suggests that IFN-I signaling may also contribute to epithelial plasticity, stress adaptation, and regenerative capacity during injury-driven repair [12,18,19,26].

Lineage tracing studies and murine intestinal organoid models have demonstrated that TA cells expand in response to injury and contribute directly to the regenerative response, often acquiring fetal-like or stress induced transcriptional states [12,20,27,28,29]. These preclinical studies further indicate that injury can trigger reversion of differentiated epithelial cells into fetal Sca1+ and Clu+ dedifferentiated epithelial states that contribute to crypt repopulation and that TA cells can transiently adopt stem-like features that enhance proliferative and regenerative capacity [27,28,29,30,31,32]. Although these mechanisms are well supported in experimental systems, their extent and functional significance in human IBD remain incompletely defined. Nevertheless, observations from human and murine inflamed colons suggest that the balance between TA and WAE states shifts toward a broader regenerative axis characterized by elevated proliferative activity but impaired terminal differentiation, with reduced mature absorptive and secretory cell populations that may compromise epithelial barrier integrity [6,12,27,28,29,30,31,32].

Consistent with this concept, single cell transcriptomic studies of human IBD tissues have identified an expansion of undifferentiated intestinal epithelial populations exhibiting heightened activity of regenerative, inflammatory, and stress response pathways. These epithelial subsets include secretory progenitors marked by Regenerating islet derived family member 4 (REG4) and stress responsive epithelial cells expressing Dual oxidase 2 (DUOX2) and cytokine stimulated genes, suggesting that injury associated reprogramming within transit amplifying compartments is coupled to innate immune signaling pathways that drive tissue remodeling [20,22]. In inflamed IBD tissue, lineage restricted epithelial molecular signatures become aberrantly expressed across absorptive, secretory, and progenitor populations, indicating a breakdown in epithelial cell type specificity and impaired differentiation during chronic inflammation [20,22,33,34,35]. Genes such as C-C motif chemokine ligand 20 (CCL20), Gliomedin (GLDN), DUOX2, CXCL1, Olfactomedin 4 (OLFM4), Mucin 1 (MUC1), REG4, Tumor Necrosis Factor Alpha-Induced Protein interacting protein 3 (TNIP3), S100 calcium binding protein P (S100P), and Dedicator of cytokinesis 4 (DOCK4) mark immature TA progenitor populations, consistent with widespread immune driven epithelial remodeling [20,22,33,34]. These epithelial states are associated with activation of TNF responsive pathways, IFN-I signaling, IL22 signaling, oxidative stress programs, and chemokine networks, suggesting that chronic immune activation may contribute to the persistence of wound associated and regenerative epithelial programs [26,35]. Collectively, these findings support the hypothesis that sustained inflammatory signaling disrupts epithelial differentiation and maintains a dysregulated regenerative state that perpetuates inflammation and impairs mucosal healing.

Building on these observations, in the intestinal mucosa, regenerative inflammation is defined as a transient, spatially restricted inflammatory state that couples immune activation with epithelial repair following injury (Table 1). Unlike classical inflammation, which drives pathogen clearance and may cause tissue damage, it promotes mucosal healing through coordinated TNF, IL-6/STAT3, IL-22, type I interferon, EGFR, WNT, and YAP/TAZ signaling that support epithelial survival, proliferation, migration, and crypt repair. It is characterized by wound-associated epithelial cells, fetal-like programs, and dedifferentiated stem-like states that enable regeneration at sites of injury. This response is typically self-resolving after barrier restoration, but persistence may contribute to chronic inflammation in IBD. It represents a coordinated immune-stromal-epithelial process and is distinct from wound-associated epithelium, fetal-like regeneration, inflammatory memory, and stress-induced stem cell states, which are component processes.

## 3. TNF as a Modulator of Epithelial Regeneration and Remodeling

Recent organoid-based and in vivo injury studies demonstrate that TNF, beyond its pro-inflammatory role, is increasingly recognized as a critical regulator of epithelial regeneration and mucosal healing, coordinating multiple signaling pathways that control epithelial survival, proliferation, stem cell plasticity, migration, and restoration of barrier integrity following intestinal injury. In murine colitis models, TNF receptor 2 (TNFR2) expression peaks in crypt epithelial cells at wound margins following Sca1 or Ly6A induction, and loss of TNFR2 prolongs Ly6a expression, sustains hyperproliferation, and delays goblet and secretory lineage differentiation, effects that are also reproduced in organoid systems [11]. Consistent with these experimental findings, TNF has been shown to promote expansion of Reg4^+^ secretory progenitors in murine and human colitis, linking inflammatory signaling to epithelial lineage remodeling and suggesting that regenerative pathways can be co-opted in chronic disease states [20,22,32]. Notably, DSS-induced colonic injury was more severe in Tnf knockout mice, indicating a role for TNF in epithelial repair processes [36]. In intestinal organoids and murine gut models, TNF has been reported to activate Wnt signaling, supporting epithelial regeneration through engagement of canonical Wnt-dependent repair pathways. These preclinical studies suggest that this occurs in part through coordinated nuclear factor kappa B (NFKB) and PI3K signaling, which cooperate with Wnt signaling to enhance β-catenin activation and drive regenerative epithelial responses [37]. TNF has also been shown in experimental systems to enhance epithelial wound healing by upregulating platelet activating factor receptor (PAFR), which activates EGFR (epidermal growth factor receptor), Src (proto-oncogene tyrosine-protein kinase Src), and Rac1 (Rac family small GTPase 1) signaling pathways to drive epithelial migration and wound closure [38]. Inhibition of TNF or loss of PAFR delayed mucosal healing in these models, supporting a regenerative role for TNF-dependent PAFR signaling during intestinal injury.

In contrast, studies using CD-derived intestinal organoids indicate that TNF can impair LGR5+ intestinal stem cell function by reducing self-renewal and organoid-forming capacity. This has been associated with disrupted Wnt/β-catenin signaling and activation of stress and inflammatory pathways, suggesting that TNF-driven inflammation may compromise epithelial regeneration by targeting the stem cell compartment in disease-relevant systems [47]. This impairment has been partially reversed in organoid models by exogenous prostaglandin E2 (PGE2), which restored stem cell expansion and organoid-forming efficiency and synergized with low-dose TNF blockade to improve epithelial regenerative capacity, although translation to in vivo human disease remains to be established. Further, in AOM/DSS colitis-associated cancer models, TNF^ΔARE^ mice, and IL10/TNF double deficient mice, experimental evidence indicates that TNF can promote tumorigenesis by sustaining chronic inflammation, epithelial hyperproliferation, and NFKB-mediated survival signaling, thereby driving hyperplasia, dysplasia, and progression to colitis-associated cancer [48]. Collectively, these experimental findings identify TNF as an injury-associated cytokine that regulates epithelial repair and intestinal stem cell plasticity, coordinating regenerative pathways that promote wound healing, progenitor expansion, and barrier restoration following injury, while suggesting that chronic TNF signaling may contribute to maladaptive or dysregulated regenerative states in persistent inflammation [11,12,20,26,35,38].

## 4. Type I Interferons and Intestinal Epithelial Homeostasis and Repair

IFN-Is are a family of cytokines, including multiple IFNα subtypes and IFNβ, that are rapidly induced in response to viral infection, cellular stress, and tissue injury to coordinate antimicrobial defense, inflammatory signaling, and immune regulation [10,14]. IFN-Is are widely recognized as context-dependent regulators of epithelial biology, with established roles in both tissue protection and inflammatory pathology depending on the timing, magnitude, and persistence of signaling during inflammation [10,14,16,17,39]. Under homeostatic and acute injury conditions, IFN-I signaling supports epithelial barrier integrity, antimicrobial defense, and controlled epithelial turnover by promoting tight junction formation, regulating proliferation, restricting excessive apoptosis, and maintaining immune tolerance toward commensal microbes. Within the intestinal epithelium, IFN-I signaling regulates stem and progenitor cell dynamics through STAT1, p21/CDKN1A, TP53/p53, and Wnt-associated signaling networks, thereby contributing to balanced proliferation, differentiation, and barrier restoration [14,40].

In contrast, preclinical and translational studies suggest that persistent or excessive IFN-I signaling in chronic inflammatory settings may be pathogenic [8,9,16,41]. In viral infection models, sustained IFN-I signaling has been shown to promote gut epithelial turnover and tissue repair through immune-epithelial crosstalk [42]. These experimental systems demonstrate that IFN-I can act on macrophages to induce regenerative mediators, including apolipoprotein L9a and L9b (Apol9a/b), which subsequently activate ERK signaling in epithelial cells to enhance proliferation and wound repair, highlighting a mechanistic axis linking immune sensing to epithelial restitution [42]. At the same time, prolonged IFN-I exposure in experimental systems has been associated with amplification of interferon-stimulated gene programs, reinforcement of NFκB-driven cytokine and apoptotic signaling, and impaired epithelial plasticity and regenerative resolution [42]. Persistent IFN-I signaling has further been linked in preclinical models to epithelial stress states, dedifferentiation, stem cell depletion, and hyperplasia associated with impaired mucosal healing [40,41,42,43].

Genetic and injury models further refine these context-dependent effects. In intestinal epithelial cell (IEC)-specific Interferon alpha and beta receptor subunit 1 (Ifnar1) deficient mice, epithelial hyperproliferation and increased Paneth cell numbers were observed relative to wildtype littermates [43]. Although these mice did not develop spontaneous inflammation or increased DSS colitis severity, they exhibited increased tumor burden in the AOM/DSS model, with effects dependent on microbiota composition, as co-housing abrogated genotype differences. These findings suggest that epithelial IFN-I signaling regulates Paneth cell function and epithelial renewal while also shaping host–microbiota interactions, with disruption of this axis potentially predisposing to dysregulated proliferation and tumorigenesis [43]. In a complementary murine model of Casein kinase 1 alpha (CK1α) deletion, IFN-I signaling was shown to restrain β-catenin-driven epithelial proliferation via activation of p53-dependent apoptosis and senescence programs, whereas combined loss of CK1α and Ifnar1 resulted in severe hyperplasia and barrier failure [49], further supporting a context-dependent restraining role of IFN-I under oncogenic stress conditions. In acute injury models, mice lacking both type I and III IFN receptors exhibit worsened epithelial injury following DSS exposure, including increased barrier disruption, goblet cell depletion, and reduced epithelial proliferation [40]. This impaired repair phenotype is associated with reduced amphiregulin (AREG) expression, an IFN-inducible mediator of epithelial regeneration [40].

Collectively, these findings support IFN-I as a context-dependent regulator of epithelial repair, integrating antimicrobial defense with epithelial turnover and immune-epithelial communication during injury, while potentially contributing to maladaptive epithelial remodeling in chronic inflammation. We propose that IFN-I functions as a context-dependent regulator of epithelial repair, where transient signaling promotes barrier restitution and immune-epithelial coordination, whereas sustained IFN-I activity in chronic intestinal inflammation may contribute to maladaptive regenerative states, impaired epithelial resolution, and altered susceptibility to IBD-associated pathology.

## 5. TNF-IFN Crosstalk and the Emergence of “Regenerative Inflammation”

Most studies investigating TNF and IFN-I crosstalk have been performed in vitro and in murine models of immune cells, particularly macrophages (reviewed in [49]), and comparatively little is known about how these synergistic pathways operate within the intestinal epithelium. In murine primary macrophages in vitro, TNF signaling has been shown to initiate a sequential inflammatory amplification loop in which early NFκB and mitogen activated protein kinase (MAPK) activation induces Interferon regulatory factor 1 (IRF1) expression, leading to low-level IFN-β production [44,45]. Autocrine IFN-β signaling subsequently activates JAK-STAT pathways, particularly STAT1, which cooperates with TNF-induced signaling to sustain chemokine expression, amplify inflammatory transcriptional mediators, and enhance expression of additional IFN-I responsive genes including IKKε, IRF7, and STAT1 [44,45,46]. Collectively, these experimental findings support a mechanistic framework in which TNF-IFN-I synergy sustain prolonged inflammatory activation and biased cellular responses toward persistent immune signaling in immune cells. However, whether an equivalent circuit operates within the human intestinal epithelium remains incompletely defined.

Preclinical studies suggest that TNF may promote early regenerative responses by stimulating epithelial proliferation, survival, and chemokine production, whereas IFN-I signaling may amplify and stabilize inflammatory and repair-associated programs. Together, these pathways may form a feed-forward inflammatory circuit that sustains epithelial activation. Recent experimental studies in human IBD organoid systems further support this model, demonstrating that combining TNF and IFN-I stimulation induces greater chemokine production, epithelial stress responses, and cytotoxicity than either cytokine alone [50], although the in vivo relevance and persistence of this interaction in human disease remains to be fully established. Collectively, the evidence supporting TNF-IFN-I crosstalk in intestinal epithelial biology is derived from complementary experimental systems with distinct limitations. Murine and macrophage-based studies provide mechanistic insight into intracellular signaling cascades and inflammatory amplification loops, while organoid systems allow controlled interrogation of epithelial responses to combined cytokine exposure. In contrast, human IBD datasets, including single-cell RNA-seq and bulk transcriptomic analyses, primarily provide correlative evidence of co-activation of inflammatory and regenerative programs without direct causal resolution. As such, the integrated model of regenerative inflammation proposed here represents a synthesis of mechanistic preclinical findings and human observational data and requires further validation in longitudinal human epithelial studies.

**Figure 1:**
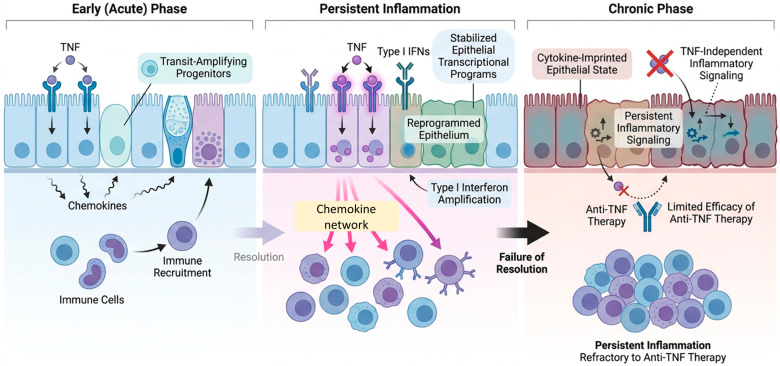
TNF-IFN crosstalk drives regenerative inflammation and therapeutic resistance in IBD. During acute injury, TNF supports epithelial repair and barrier restoration, but in chronic disease, persistent TNF and type I interferon signaling drive sustained inflammatory epithelial activation, creating a self-reinforcing regenerative inflammation circuit that may contribute to anti-TNF resistance.

These findings support a model in which TNF and IFN-I signaling are dynamically reprogrammed across phases of epithelial injury and inflammation. During acute injury, coordinated TNF and IFN-I signaling may support mucosal repair through progenitor expansion, transient chemokine production, and restoration of epithelial barrier integrity (Figure 1). In contrast, in chronic inflammatory settings, sustained TNF and IFN-I activity may contribute to impaired resolution by maintaining inflammatory chemokine expression, altering apoptotic signaling pathways, and reinforcing regenerative inflammatory programs. We propose that this may establish a self-reinforcing epithelial–immune circuit, termed regenerative inflammation, in which repair-associated pathways become dysregulated and contribute to persistent disease rather than restoration of homeostasis. As these programs become progressively stabilized, epithelial activation may become partially independent of TNF signaling, although direct evidence for this in human intestinal epithelium remains limited and is currently inferred from preclinical and organoid-based studies. We propose that TNF-IFN crosstalk is dynamically reprogrammed across phases of intestinal injury (Figure 1). During acute inflammation, coordinated TNF and IFN-I signaling supports epithelial repair and barrier restoration. In contrast, during chronic disease, persistent activation of these pathways may sustain inflammatory chemokine production, impair epithelial differentiation, and lock epithelial cells into activated, poorly differentiated states associated with ongoing immune recruitment and tissue remodeling. We refer to this persistent injury response as regenerative inflammation, in which repair programs become dysregulated and contribute to chronic disease progression. As these regenerative programs become progressively stabilized, we hypothesize that they may become less dependent on TNF signaling alone, potentially contributing to therapeutic resistance in IBD. Evidence from experimental models demonstrates that TNF and IFN-I signaling can cooperatively regulate epithelial plasticity, wound healing responses, and chemokine production; however, whether similar mechanisms operate in the intestinal epithelium of anti-TNF non-responders remains to be established.

## 6. Regenerative Inflammation and Anti-TNF Response

Persistent TNF-IFN-I crosstalk in the intestinal epithelium under chronic inflammatory conditions may contribute to a sustained regenerative inflammatory state, which has been proposed as a potential contributor to variable responses to anti-TNF therapy in IBD. While TNF blockade is effective in many patients, studies in human IBD tissues and experimental models suggest that IFN-I activity may be associated with maintenance of epithelial regenerative and stress response programs, as well as chemokine expression. However, a causal role for IFN-I in sustaining inflammation independently of TNF in human disease has not yet been demonstrated. Clinically, epithelial regenerative and stress-associated states have been observed in cohorts overlapping with anti-TNF non-responders, where inflammation persists despite TNF inhibition, although causality in human disease remains unproven. These regenerative inflammatory programs have been proposed to not only reflect disease severity but also actively drive tissue remodeling, alter differentiation trajectories, and increase epithelial sensitivity to apoptotic and senescence cues, potentially contributing to fibrosis or tumorigenesis. Preclinical and translational studies, including patient-derived organoids and single-cell analyses, show that epithelial cells from non-responders display combined proliferative plasticity and stress adaptation signatures [20,22,27,47], suggesting anti-TNF response may be influenced by epithelial-intrinsic programs modulated by IFN-I signaling, although this remains to be established in vivo. We propose that regenerative epithelial states contribute to anti-TNF non-response by sustaining stress-adaptive and proliferative programs despite TNF blockade, thereby reinforcing chronic inflammation and impaired mucosal resolution.

## 7. Therapeutic Implications and Conclusions

Recognition of regenerative inflammation as a central feature of IBD may help to broaden therapeutic strategies beyond conventional immunosuppression. Targeting interferon signaling, for example, via JAK-STAT pathway inhibition, has been proposed as a potential approach to modulate epithelial stress and repair programs, with the possibility of influencing responsiveness to anti-TNF therapy in some patient subsets, although clinical efficacy of such strategies remains to be established. Combination approaches that modulate both TNF and IFN-I associated pathways may be of interest to interrupt potentially self-perpetuating inflammatory circuits, although this remains to be demonstrated in clinical studies.

Epithelial-derived markers, including REG4, DUOX2, CXCL1, and CXCL10, have been consistently identified in patient-derived and single-cell transcriptomic studies as features of regenerative and inflammatory epithelial states in IBD. However, current evidence is largely derived from cross-sectional profiling rather than longitudinal, therapy-stratified cohorts, and does not support validated predictive performance for anti-TNF response. These markers should therefore be interpreted as indicators of dynamic epithelial injury and repair programs, reflecting active inflammatory states rather than stable patient endotypes. Their expression is consistent with ongoing epithelial–immune activation rather than established classifiers of therapeutic outcome. This distinction is important, as epithelial transcriptional programs are highly plastic and influenced by cytokine milieu, disease activity, and microbiome context, limiting their reliability as standalone predictive biomarkers. Nevertheless, these candidate markers may provide biological insight into regenerative inflammatory states and could inform future stratification strategies, pending prospective validation in treatment-response cohorts.

Organoid-based approaches may further support functional characterization of regenerative inflammatory states and could provide a platform for exploratory patient stratification, including identification of individuals potentially less likely to respond to anti-TNF therapy, thereby informing hypothesis-generating approaches to more personalized treatment strategies, pending prospective clinical validation.

Overall, regenerative inflammation may represent a useful conceptual framework for integrating epithelial biology into therapeutic decision-making alongside classical immune targeting. Future strategies will likely require a better understanding of epithelial–immune interactions and the balance between regeneration and resolution, rather than focusing solely on suppression of inflammatory signaling. In conclusion, IBD pathogenesis involves both immune dysregulation and altered epithelial regeneration. TNF-associated repair pathways are important for acute mucosal healing but may become maladaptive in the context of sustained inflammatory signaling and interferon-associated epithelial stress. This chronic epithelial state, characterized by persistent activation and chemokine production, may contribute to therapeutic non-response in some patients. Integrating experimental, organoid, and clinical observations provides a framework for future studies aimed at improving stratified and mechanism-based approaches in IBD.

## Figures and Tables

**Table 1:** Comparative features of homeostatic regeneration, regenerative inflammation, and classical inflammation in intestinal epithelial biology.

Feature	Homeostatic Regeneration	Regenerative Inflammation	Classical Inflammation
Primary function	Routine epithelial turnover [5,6,29]	Tissue repair and barrier restoration after injury [12,19,26,27]	Pathogen elimination and immune defence [17,35]
Trigger	Physiological cell loss [5,29]	Epithelial injury, ulceration, barrier disruption [12,26,27]	Infection, injury, microbial invasion [17,36]
Dominant epithelial state	LGR5+ stem cell renewal [4,29,32]	Wound-associated epithelial states, fetal-like reversion, dedifferentiation [12,27,30,33,34]	Stress-associated and inflammatory epithelial states [22,24]
Key signaling pathways	WNT, Notch, BMP [5,6]	TNF, IL-22, IL-6/STAT3, IFN-I, EGFR, YAP/TAZ, WNT [11,12,14,19,26,37,38,39,40,41,42,43]	TNF, IL-1β, IL-6, NFκB, IFNγ [17,35,36,44,45,46]
Stem cell behavior	Normal self-renewal [29,31]	Stem cell activation and epithelial plasticity [27,30,34]	Frequently impaired or dysregulated [24,47]
Spatial location	Crypt base [29,32]	Ulcer margins and regenerating crypts [12,27,33]	Diffuse inflamed tissue [22,24]
Duration	Continuous [29]	Transient and self-resolving repair programs [12,19]	Acute or chronic [17,48]
Outcome	Homeostasis [5,6]	Mucosal healing and barrier restoration [12,19,26]	Tissue injury, chronic inflammation, and pathological remodeling [17,48]
Relevance to IBD	Maintains epithelial integrity [4,5]	Associated with mucosal healing, epithelial plasticity, and transcriptional states linked to treatment response [21,25,34]	Drives disease activity and tissue damage [2,3,36,48]

## Data Availability

No new data were created or analyzed in this study.

## Abbreviations

The following abbreviations are used in this manuscript:

IFN-I	Type I Interferon
TNFR	Tumor Necrosis Factor Receptor
TNF	Tumor Necrosis Factor
IBD	Inflammatory Bowel Disease
RNA-Seq	RNA Sequencing
CD	Crohn’s Disease
NR-CD	Non-Responsive CD
R-CD	Responsive CD
IEC	Intestinal epithelial cells
IFNs	Interferons
scRNA-Seq	Single cell RNA Sequencing
TNFR1	TNF receptor 1
Wnt	Wingless related integration site
TA cells	Transit amplifying cells
WAE	Wound-associated epithelium
KRT6	Keratin 6
KRT17	Keratin 17
CLU	Clusterin
SCA1	Stem cell antigen 1
TACSTD2/TROP2	Tumor associated calcium signal transducer 2/Trophoblast cell surface antigen 2
ANXA1	Annexin A1
CXCL1	Chemokine (C-X-C motif) ligand 1
CXCL10	Chemokine (C-X-C motif) ligand 10
CXCL11	Chemokine (C-X-C motif) ligand 11
YAP/TAZ	Yes-associated protein/Transcriptional coactivator with PDZ-binding motif
IL6	Interleukin 6
REG4	Regenerating islet derived family member 4
DUOX2	Dual oxidase 2
CCL20	C-C motif chemokine ligand 20
GLDN	Gliomedin
OLFM4	Olfactomedin 4
MUC1	Mucin 1
TNIP3	Tumor Necrosis Factor Alpha-Induced Protein interacting protein 3
S100P	S100 calcium binding protein P
DOCK4	Dedicator of cytokinesis 4
TNFR2	TNF receptor 2
PAFR	Platelet activating factor receptor
EGFR	Epidermal growth factor receptor
Src	Proto-oncogene tyrosine-protein kinase Src
Rac1	Rac family small GTPase 1
PGE2	Prostaglandin E2
AOM/DSS	Azoxymethane/dextran sulfate sodium
STAT1	Signal transducer and activator of transcription 1
p21/CDKN1A	Cyclin dependent kinase inhibitor 1A
TP53/p53	Tumor protein p53
Apol9a/b	Apolipoprotein L9a and L9b
ERK	Extracellular signal regulated kinase
IEC	Intestinal epithelial cell
Ifnar1	Interferon alpha and beta receptor subunit 1
CK1α	Casein kinase 1 alpha
AREG	Amphiregulin
NF-κB	Nuclear factor kappa B
MAPK	Mitogen activated protein kinase
IRF1	Interferon regulatory factor 1
